# Microbial Biofilms, Second Edition

**DOI:** 10.3201/eid2206.160282

**Published:** 2016-06

**Authors:** Rodney M. Donlan

**Affiliations:** Centers for Disease Control and Prevention, Atlanta, GA, USA

**Keywords:** Microbial biofilm, bacterial biofilm, fungal biofilm, biofilm methods, genomic, metagenomic, transcriptomic, proteomic, biofilm tolerance, antimicrobial resistance

Microbial Biofilms provides an overview of the formation, structure/architecture, cell-to-cell interactions, and dispersal of fungal and bacterial biofilms. The target audience is biofilm researchers, but this second edition of the book should also be useful for healthcare practitioners seeking a better understanding of microbial biofilms in healthcare delivery.

The number of published papers pertaining to microbial biofilms in healthcare and public health has continued to grow since the publication of the first edition of Microbial Biofilms in 2004. For example, a search using PubMed for 2004–2016 identified 488 publications when using the search terms “biofilm and healthcare-associated infection” and 7,303 publications using the search terms “biofilm and public health,” compared to 73 and 1,459 publications, respectively, for 1992–2003. The current edition addresses this level of interest; several of the contributions in this book specifically focus on the role of biofilms in disease processes (Chapters 6, 7, 8, 14, and 19) or biofilm susceptibility to antimicrobial agents (Chapters 2 and 13).

Chapter 1 provides a balanced comparison of static and continuous flow methods for growing biofilms that should be beneficial for researchers investigating biofilm development or dispersion, and for applied studies evaluating new treatment strategies for biofilm prevention and control. This beginning can enable those new to the field to evaluate the benefits and drawbacks of different biofilm testing methods for specific applications. Chapter 1 also provides a helpful but brief discussion of the use of “omic” approaches (i.e., genomic, metagenomic, transcriptomic, and proteomic) in the study and characterization of biofilms.

Protocols to evaluate biofilm control strategies in vivo are needed, since in vitro methods may not predict performance under the more robust conditions provided in an animal model (*1*). Published animal model protocols for evaluating biofilm control are few. Chapter 3 provides several animal models for the evaluation of fungal biofilms (primarily *Candida* spp.), including vascular catheter, urinary catheter, and subcutaneous implant model systems. However, animal model systems for the characterization of bacterial biofilms are not described.

Several recent papers have used culture-independent methods to characterize biofilms on indwelling medical devices (*2*–*4*). With the exception of a brief discussion in Chapter 1, very little information is provided on the benefits and drawbacks of culture-independent methods to characterize clinically relevant biofilm communities.

Chapter 13 provides an excellent overview of antimicrobial tolerance in biofilms, with a good summary of the factors that can influence susceptibility. I found particularly helpful the use of tolerance factors to compare reduced susceptibility of different biofilm-associated organisms toward biocides, antiseptics, and antibiotic drugs. Tolerance factors were plotted as a function of antimicrobial agent molecular weight, substratum material, and biofilm density, providing the reader a method for quickly visualizing these patterns for a wide range of organisms. This information can be very helpful when developing experimental approaches to evaluate biofilm control strategies.

In summary, Microbial Biofilms is a useful compendium suitable for students and a practical guide for researchers investigating new biofilm treatment strategies. The emphasis on the role of biofilms in the pathogenesis of various microbial diseases, as well as discussions of biofilm tolerance and antimicrobial resistance should also be helpful and interesting to anyone working in the field of healthcare delivery.
